# Comparison of Malaria Simulations Driven by Meteorological Observations and Reanalysis Products in Senegal

**DOI:** 10.3390/ijerph14101119

**Published:** 2017-09-25

**Authors:** Ibrahima Diouf, Belen Rodriguez-Fonseca, Abdoulaye Deme, Cyril Caminade, Andrew P. Morse, Moustapha Cisse, Ibrahima Sy, Ibrahima Dia, Volker Ermert, Jacques-André Ndione, Amadou Thierno Gaye

**Affiliations:** 1 Laboratoire de Physique de l’Atmosphère et de l’Océan-Siméon Fongang, Ecole Supérieure Polytechnique de l’Université Cheikh Anta Diop (UCAD), BP 5085, Dakar-Fann, Dakar 10700, Senegal; atgaye@ucad.edu.sn; 2 Department of Geophysics and Meteorology, Universidad Complutense de, Plaza de las Ciencias s/n, Madrid 28040, Spain; brfonsec@fis.ucm.es; 3 Instituto de Geociencias IGEO, CSIC-UCM, Agencia Estatal del Consejo Superior de Investigaciones Científicas, Madrid 28040, Spain; 4 Unité de Formation et de Recherche de Sciences Appliquées et de Technologie, Université Gaston Berger de Saint-Louis, BP 234, Saint-Louis 32000, Senegal; abdoulaye.deme@ugb.edu.sn; 5 Department of Epidemiology and Population Health, Institute of Infection and Global Health, University of Liverpool, Water House Building, Liverpool L693GL, UK; Cyril.Caminade@liverpool.ac.uk; 6 National Institute for Health Research [M1] (NIHR), Health Protection Research Unit in Emerging and Zoonotic Infections, Liverpool L69 3GL, UK; A.P.Morse@liverpool.ac.uk; 7 Department of Geography and Planning, School of Environmental Sciences, University of Liverpool, Roxby Building, Liverpool L69 7ZT, UK; 8 Programme National de Lutte contre le Paludisme (PNLP), BP 25 270 Dakar-Fann, Dakar 10700, Senegal; mcdoussouye@yahoo.fr; 9 Centre de Suivi Ecologique, BP 15532, Fann Résidense, Dakar 10700, Senegal; ibrahima.sy@cse.sn (I.S.); jacques-andre.ndione@cse.sn (J.-A.N.); 10 Institut Pasteur de Dakar (IPD), Unité d’Entomologie Médicale, 36 Av. Pasteur, BP 220 Dakar, Dakar 12900, Senegal; dia@pasteur.sn; 11 Institute of Geophysics and Meteorology, University of Cologne, Kerpenerstr. 13, D-50923 Cologne, Germany; vermert@meteo.uni-koeln.de

**Keywords:** climate, malaria, observations, simulations, stations, Senegal, model

## Abstract

The analysis of the spatial and temporal variability of climate parameters is crucial to study the impact of climate-sensitive vector-borne diseases such as malaria. The use of malaria models is an alternative way of producing potential malaria historical data for Senegal due to the lack of reliable observations for malaria outbreaks over a long time period. Consequently, here we use the Liverpool Malaria Model (LMM), driven by different climatic datasets, in order to study and validate simulated malaria parameters over Senegal. The findings confirm that the risk of malaria transmission is mainly linked to climate variables such as rainfall and temperature as well as specific landscape characteristics. For the whole of Senegal, a lag of two months is generally observed between the peak of rainfall in August and the maximum number of reported malaria cases in October. The malaria transmission season usually takes place from September to November, corresponding to the second peak of temperature occurring in October. Observed malaria data from the *Programme National de Lutte contre le Paludisme* (PNLP, National Malaria control Programme in Senegal) and outputs from the meteorological data used in this study were compared. The malaria model outputs present some consistencies with observed malaria dynamics over Senegal, and further allow the exploration of simulations performed with reanalysis data sets over a longer time period. The simulated malaria risk significantly decreased during the 1970s and 1980s over Senegal. This result is consistent with the observed decrease of malaria vectors and malaria cases reported by field entomologists and clinicians in the literature. The main differences between model outputs and observations regard amplitude, but can be related not only to reanalysis deficiencies but also to other environmental and socio-economic factors that are not included in this mechanistic malaria model framework. The present study can be considered as a validation of the reliability of reanalysis to be used as inputs for the calculation of malaria parameters in the Sahel using dynamical malaria models.

## 1. Introduction

Malaria is a vector-borne disease, which requires three essential factors for its existence and transmission: *Plasmodium* parasites, mosquito vectors, and human hosts [1,2]. There are three specific species of *Plasmodium* affecting humans, which are *Plasmodium falciparum* (the most common and lethal parasite species in sub-Saharan Africa), *Plasmodium vivax*, and *Plasmodium ovale*, the common species to humans and gorillas. *Plasmodium knowlesi*, like *Plasmodium malariae*, generally affects primates and gorillas, with some examples of transmission to humans in Malaysia [3]. The two major parasites are *P. falciparum* in the Tropics and *P. vivax* in more temperate regions. There are various species of anopheles vectors which are competent for malaria transmission. The main malaria vectors in Africa and Senegal are *Anopheles gambiae*, *Anopheles funestus*, *Anopheles arabiensis*, and *Anopheles melas* to a lesser extent [4,5,6]. Beyond these essential factors, malaria transmission risk can be sustained or strengthened by specific environmental and climatic conditions as well as a large range of socio-economic factors for the African countries [7,8].

In particular, the influence of climate variability on health is widely recognized [9,10]. The economic development in Africa is severely affected by a human and animal disease burden [11,12]. During the 1970s and 1980s, rainfall decreased by nearly 30% over the Sahel, with severe droughts occurring in 1972, 1983, and 1991–1992 [13,14,15,16,17]. Some studies have highlighted how the drought during the 1970s and 1980s reduced the distribution and abundance of mosquito vectors without leading to drastic malaria transmission [18]. Increasing drug resistance in children and pregnant women populations with altered immunity illustrates this finding about high malaria occurrence despite reduced rainfall in the Sahel [19]. Regarding the relationship between rainfall and malaria, some studies [20,21,22] have investigated the malaria decrease and drought in the *Niayes* (a northwestern area in Senegal) and the Sahel in general. The main malaria vector, *An. funestus*, in these regions has almost disappeared following the drought in the 1970s and 1980s [17]. The abundance of other anopheles species also decreased significantly over the region, compared to *An. arabiensis*, which is another efficient malaria vector due to its zoophilic characteristics in rural areas [23,24,25,26,27]. Thus, the observed decline of malaria is related to the disappearance of *An. funestus* and declining population of *An. gambiae*, which can be attributed to the long term drought and human activities, including deforestation [28]. However, since the early 2000s, increasing rainfall has favored the re-emergence of malaria vectors in the Sahel region, in particular for the *An. gambiae* species [29]. In some regions, such as in the Senegal River Basin, *An. funestus* has returned due to the availability of permanent man-made water ponds. In Senegal, rainfall events and increasing human activities (the building of small dams, and irrigation for agriculture) modify the environment and could still cause malaria epidemics, especially in local areas where people are highly exposed and susceptible to malaria [29].

Many studies have investigated the influence of climatic factors on the epidemiology of malaria in Senegal [30,31,32]. The results have highlighted the need to further study seasonal malaria transmission in Senegal, particularly in the Ferlo area. The Ferlo is located in the North; it is the most Sahelian part of the Senegal territory. The alternation of dry and wet seasons and the occurrence of extreme events, such as floods or droughts, can significantly impact malaria in terms of mortality as well as morbidity. Climate variability can impact malaria transmission through increased temperatures, as highlighted by [33] for Zimbabwe. Warm temperatures accelerate the life development of anopheles vectors and facilitate malaria transmission by reducing the duration of the reproductive cycle of the parasite (the so-called *sporogonic* cycle) within the mosquito vector [34,35,36]. Rainfall intensity and frequency changes during the rainy season can modulate the development of mosquito population through the availability of breeding sites [37,38,39,40].

In this study, we focus on the impact of climate variability on malaria transmission dynamics in Senegal.

We utilized the Liverpool Malaria Model version 2010 (LMM2010 or LMM hereafter) driven by different climate datasets to simulate a series of malaria incidence for Senegal. This mechanistic malaria model allows the exploration of variability related to the impact of climatic factors solely; it does not include other important socio-economic factors. The same method has been utilized by [41,42,43] to study the impact of climate variability on Rift Valley Fever and malaria. Due to the non-availability of recorded clinical malaria data over a long continuous time period, we used the LMM to simulate potential malaria parameters such as human biting rates (HBR hereafter), the entomological inoculation rate (EIR hereafter), and female mosquito abundance. These parameters are first compared to observed malaria case data for the period 2001–2010. We then ran these simulations over a longer time period (1910–2009) in order to assess low frequency changes in simulated malaria burden over Senegal.

Within the framework of Roll Back Malaria, special efforts might be achieved in Senegal in terms of malaria prevention and control in the upcoming years. To this aim, long time series are needed to understand the influence of climate variability on malaria dynamics.

The main motivations underlying this malaria modeling exercise are:

- Real malaria data are unevenly distributed within countries and across regions;
- The length of observed time series for malaria data is generally short (2001–2010 for Senegal).
- The available data does not only depend on climate, but it also relies on other socioeconomic factors. It is thus important to disentangle the influence of climate on malaria compared to other driving factors. Consequently, we aim to address the following questions:
- Where does malaria happen regularly, occasionally, and never?
- When and how does the malaria transmission season take place?

The present study uses observations for 11 particular locations in Senegal, for which clinical malaria data and meteorological observations were available. The study also takes advantage of the available reanalysis data to drive the malaria model over a larger spatial domain and a longer time period (1910–2009). The study can be considered as a validation of the use of reanalyses as inputs for the dynamical malaria model.

The PNLP clinical observations data are used to validate simulated malaria transmission in Senegal. Malaria parameters are modelled based on meteorological station data for Senegal, and the model outputs are validated with respect to clinical observations recorded by the PNLP. The PNLP is under the General Directorate of Health, which in turn is under the Direction of Disease Control in Senegal. The available period of study is extended using reanalyses temperature and satellite rainfall data to drive the malaria model backward in time. Furthermore, long-term changes in malaria burden are evaluated together with the reliability of reanalyses as driving conditions for the malaria model, using evidence from the published literature.

The observation and modelled data sets and the methodology are described in Section 2, while the results and discussions are shown in Section 3 before providing the conclusion in Section 4.

## 2. Materials and Methods

### 2.1. The Liverpool Malaria Model

The LMM is a dynamical malaria model driven by daily time series of rainfall and temperature. The various components of the malaria transmission model and the parameter settings are further described by [44,45]. The LMM is a mathematical–biological model of parasite dynamics, which comprises the weather-dependent within-vector stages and the weather-independent within host stages. The mosquito population is simulated using larval and adult stages, with the number of eggs deposited into breeding sites and the larval mortality rate depending on the previous 10 days’ rainfall. The adult mosquito mortality rate and the egg-laying/biting cycle (so-called *gonotrophic* cycle) also depend on temperature. The process of parasite transmission between humans and mosquitoes is modelled with temperature dependencies for the replication rate of the parasite within the mosquito (*sporogonic* cycle) and the mosquito biting rate. Both cycles evolve as a function of the number of ‘degree days’ above a specific temperature threshold. Respectively, the *gonotrophic* and the *sporogonic* cycles take approximately 37 and 111 degree days with a threshold of 9 °C (18 °C) [43]. The model does not simulate host immunity. The LMM is very sensitive to the climate data inputs and the disease model parameterization. Studies about climate and health have used LMM simulations in southern Africa, including Zimbabwe, Botswana, and for the whole African continent [46,47].

The parameters provided as model outputs are: the Entomological Inoculation Rate (EIR), which represents the number of infectious bites per person per month, the Human Biting Rate (HBR), which represents the total number of bites per person per month, and the total number of female mosquitoes (Nm hereafter).

The LMM uses a set of parameters and different egg, larval, and mosquito survival schemes based on the literature and Ph.D. studies in West Africa. This model version (LMM2010) showed significant improvements in simulating malaria dynamics over Sub-Saharan Africa countries, including Senegal. This version was also employed by [48,49,50] to assess the risk posed by future climate change on malaria burden. In this study, we employed this version of parameter sets for the malaria model (see Table S1 for further details about the parameter setting).

### 2.2. Observed Datasets

**Scheme 1:**
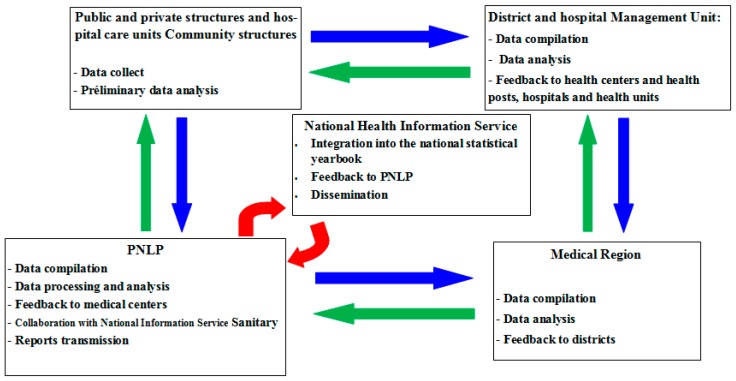
Description of the *Programme National de Lutte contre le Paludisme* (PNLP) network covering the public health centres in Senegal. The different steps consist in collecting and processing the data, analyzing the results, and disseminating governmental reports.

Malaria simulations driven by both meteorological station and reanalysis data are carried out using the LMM. The evolution of malaria model outputs is then compared with the evolution of observed malaria cases obtained from the PNLP. The PNLP records malaria cases in the framework of field survey in Senegal. The description of the PNLP network in Senegal is shown in Scheme 1. 

All age groups are screened in the PNLP data set. The observed numbers of malaria cases are available for various health districts in Senegal for the period 2001–2010. This malaria data is recorded for all health districts and hospitals to derive monthly time series for 11 main stations (Dakar, Diourbel, Fatick, Kaolack, Kolda, Louga, Matam, Saint-Louis, Tambacounda, Thies, and Ziguinchor). The selection of these sentinel sites provides a good representation of malaria transmission in the different climatic zones of Senegal [51,52,53,54,55].

The LMM outputs driven by meteorological data are also analyzed. Temperature data are obtained from different reanalysis datasets. The sources of these reanalysis datasets are: the National Oceanic Atmospheric Administration (NOAA) 20th Century reanalysis, the National Centers for Environmental Prediction (NCEP), and the European Center for Medium Range Weather Forecast reanalysis (ERA40 and ERA Interim). These daily temperature based on different reanalysis datasets are used to drive the LMM: the 20th century reanalysis (20th CR hereafter, [56]), NCEP [57], ERA40 [58], and ERA Interim data [59]. Daily rainfall data comes from the Climate Hazards Group InfraRed Precipitation with Stations (CHIRPS) [60] calibrated satellite product, as reanalysis rainfall was not judged as a reliable variable. The 20th century reanalysis covers the period 1910–2009 at a 2.5° × 2.5° spatial resolution; NCEP (National Centers for Environmental Prediction) reanalysis covers the period 1960–2009 with the same spatial resolution.

ERA40 reanalysis data is available for the period 1958–2001 at a 2.5° × 2.5° spatial resolution, and ERA Interim reanalysis data is available for the period 1979–2011 at a 1.5° × 1.5° spatial resolution. CHIRPS rainfall data covers the 1981–2016 period at a 0.25° × 0.25° spatial resolution. The CHIRPS product was carried out by the U.S. Geological Survey (USGS) in collaboration with the University of California, Santa Barbara, Climate Hazards Group. We also describe malaria variability driven by the 20th Century Reanalysis data in order to characterize long-term changes in simulated malaria dynamics over Senegal. 

We aim to characterize the spatio-temporal variability of simulated malaria parameters, such as [61] that used in the seasonal climate forecast from the ENSEMBLES Project [62] to drive the LMM over a common period.

**Figure 1:**
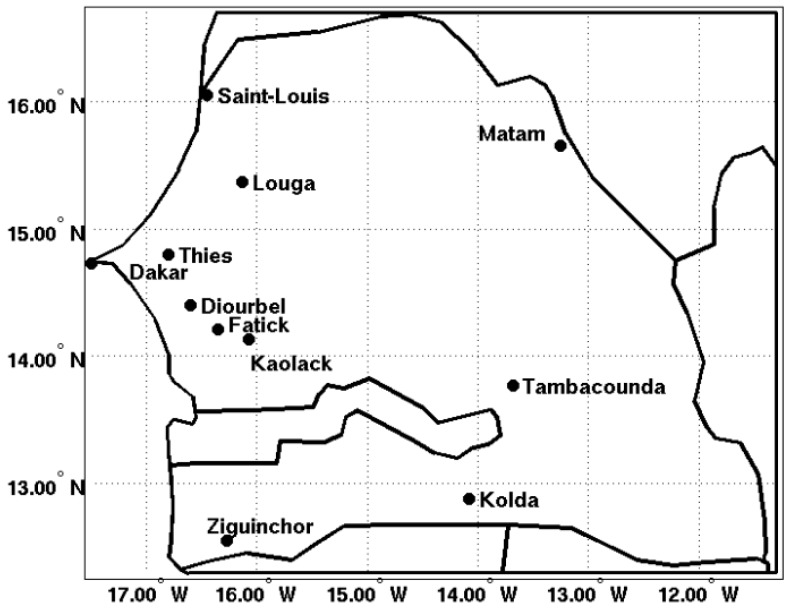
Meteorological and malaria observation stations. The map shows the locations of the stations used in this study. The stations in alphabetical order are: Dakar (14.73° N, 17.5° W), Diourbel (14.4° N, 16.6° W), Fatick (14.21° N, 16.35° W), Kaolack (14.13° N, 16.07° W), Kolda (14.88° N, 14.08° W), Louga (15.37° N, 16.13° W), Matam (15.65° N, 13.25° W), Saint-Louis (16.05° N, 16.45° W), Tambacounda (13.77° N, 13.68° W), Thies (16.05° N, 16.45° W), and Ziguinchor (12.55° N, 16.27° W).

We extracted reanalysis temperature and rainfall data and CHIRPS rainfall estimates for 11 stations of Senegal: Dakar (14.73° N, 17.5° W), Diourbel (14.4° N, 16.6° W), Fatick (14.21° N, 16.35° W), Kaolack (14.13° N, 16.07° W), Kolda (14.88° N, 14.08° W), Louga (15.37° N, 16.13° W), Matam (15.65° N, 13.25° W), Saint-Louis (16.05° N, 16.45° W), Tambacounda (13.77° N, 13.68° W), Thies (16.05° N, 16.45° W), and Ziguinchor (12.55° N, 16.27° W). These stations are representative of different eco-climatic zones of Senegal. Spatial averages of LMM outputs are calculated for Saint-Louis and Louga and Matam (North and Northeast), Dakar, Diourbel, Fatick, Thies, and Kaolack (Center), and Tambacounda, Kolda, and Ziguinchor (South and Southeast). The Senegal domain has been divided into three areas: the southern, central, and northern zones. A spatial average is also carried out for all stations at the country level. The area of study is shown in Figure 1. Table 1 summarizes the different climatic and health datasets used in this study.

## 3. Results

### 3.1. Climate Context

**Figure 2:**
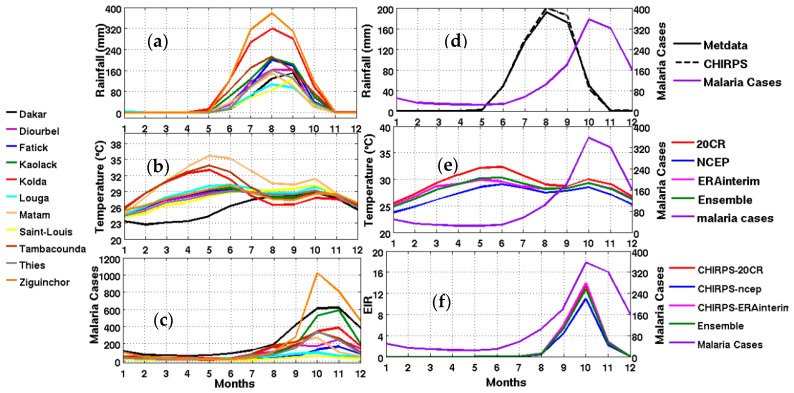
Left panel: (**a**) observed rainfall in mm; (**b**) averaged reanalysis temperature in °C (20th century reanalysis (20CR), NCEP, and ERA Interim); (**c**) mean annual cycle of observed malaria cases for different Senegalese stations from 2001 to 2010 corresponding to the common available period of observed rainfall and reanalyses data (20CR, NCEP, and ERA Interim and the ensemble mean of reanalyses); Right panel: Mean annual cycle of climate parameters, the simulated malaria parameter, and observed malaria cases: (**d**) rainfall; (**e**) temperature; (**f**) Entomological Inoculation Rate (EIR) (common period 2001–2010) and observed malaria cases in averaged Senegal stations (right y-axis for each figure).

Climate characteristics are very diverse across different eco-regions of Senegal, ranging from a Sahelian to a Sudano-Sahelian and a Sudano-Guinean climate. Senegalese climate is strongly modulated by the West African monsoon [63]. In the northern part of Senegal, the climate is Sahelian, with moderate rainfall occurring during a very short rainy season (August–September). Due to its northernmost position, the station of Saint-Louis located in the River Valley of Senegal (*Vallée du Fleuve Sénégal*), is characterized by a very dry climate compared to the southern part of Senegal. The monthly amount of rainfall is less than 160 mm (Figure 2b). In Saint-Louis, the rainy season takes place generally between June and October, with a maximum rainfall observed in September. The average monthly temperatures are moderate due its coastal position, but the temperatures can reach up to more than 29 °C during summer (Figure 2c).

The Ferlo (Louga) experiences a Sudano-Sahelian climate. The rainfall maximum is observed in August, with slightly larger values than Saint-Louis during this month. The difference found in terms of rainfall (Figure 2a) between stations is also consistent with the temperature features. The period from December through February is marked by lower temperatures (Figure 2b).

Except Dakar and Saint-Louis, due to their coastal position, the stations of Matam, Tambacounda, and Diourbel present a bimodal evolution of seasonal temperature, with two peaks in May and October, either side of the rains. The bimodal evolution appears also for the rest of the stations, but it is less marked.

Dakar is located at the westernmost tip of West Africa. Low temperatures and high humidity values are found due to sea breeze effects. Dakar, Diourbel, Fatick, Kaolack, and Thies are all located in Central Senegal, but there is a significant difference in rainfall and temperature peaks in August (Figure 2b), as Diourbel, Fatick, and Kaolack are located in the *Groundnut Basin* (“*Bassin arachidier*” or “*Sine Saloum*”). A long and dry season (9 months) is followed by the rainy season, which usually begins in June–July and ends in early October. The Central part of Senegal generally experiences a tropical sudanese climate. However, it can range from a Sudano-Sahelian climate in the northern part, where rainfall ranges between 400 and 600 mm, to a Sahelian climate in the southern part, where rainfall ranges between 600 mm and 800 mm. In the “*Groundnut Basin*”, temperatures are high from April to June and from September to November, with two peaks occurring in May and October (around 30 °C, Figure 2b).

The east of Senegal is one of the warmest regions of the country during the dry season. During the rainy season, the temperature decreases significantly due to land surface cooling associated with larger precipitation. This region is generally represented by the Tambacounda station, and its climate ranges between Sudano-Sahelian and Sudano-Guinean. The rainfall maximum is recorded in August; it can reach up to 200 mm during this month. The rainy season lasts 4 to 5 months, and it usually starts in May–June and ends in October (Figure 2a). The relative humidity is consistently high during the rainy season, peaking between August and October (data not shown).

The southern part of the country, mainly represented by Ziguinchor and Kolda (“*Basse-Casamance*”), records the largest amount of rainfall in Senegal. The vegetation cover is characterized by predominant savannah. This region is characterized by a Sudano-Guinean climate, with a 4- to 6-month duration of the rainy season. The temperatures are lower compared to the North. This southern area is considered as the gateway generator of important humid winds; it is the wettest region in Senegal, with a longer rainy season that ranges from May to November (Figure 2a). The peak of rainfall is recorded in August (more than 350 mm), and it is almost equivalent to the annual rainfall observed in the North of the country. For other regions, the monsoon onset usually starts in June.

In summary, the largest temperature values are observed in eastern stations, such as Matam and Tambacounda, but also in Kolda, which is located in the southeastern region. Lower temperatures are shown in the western, central, and southwestern areas. The southern and southeastern parts record lower temperatures due to heavy precipitation during the rainy season i.e., September–October–November.

**Figure 3:**
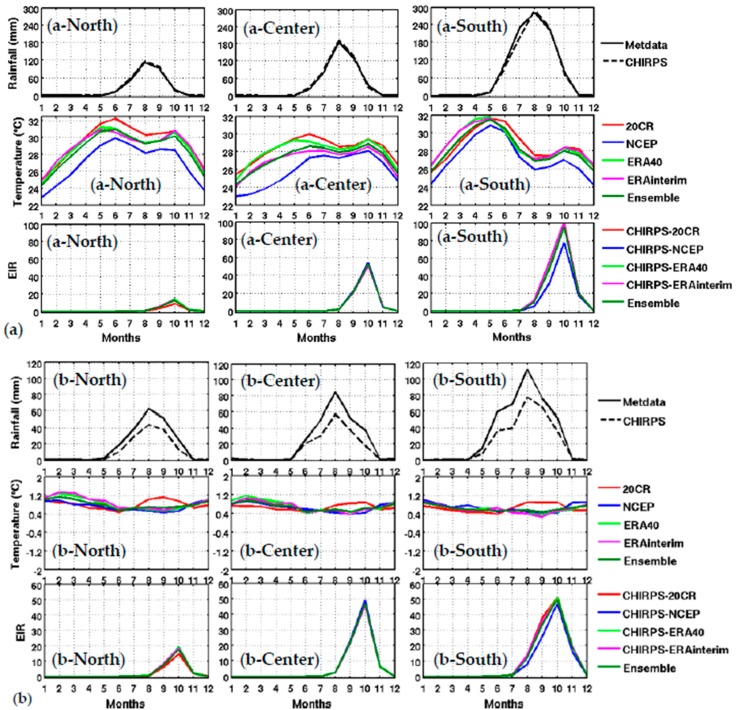
Upper panel: Mean annual cycle of climate parameters and the simulated malaria parameter: (**a**) Meteorological rainfall data based on weather stations (Metdata) and CHIRPS satellite rainfall (CHIRPS), reanalysis temperature (20CR, NCEP, ERA40, and ERA Interim) and simulated EIR based on CHIRPS rainfall and reanalysis temperature (CHIRPS-20CR, CHIRPS-NCEP, CHIPRS-ERA40, CHIRPS-ERA Interim, and the ensemble mean of all reanalyses), for different Senegal zones (northern in column 1: spatial average for Saint-Louis, Louga, and Matam), central in column 2 (spatial average for Dakar, Thies, Kaolack, Fatick, and Diourbel) and southern in column 3 (spatial average for Ziguinchor, Kolda, and Tambacounda) for the period 1981–2001. Lower panel: (**b**) idem as the upper panel, but for the standard deviations (SD).

**Figure 4:**
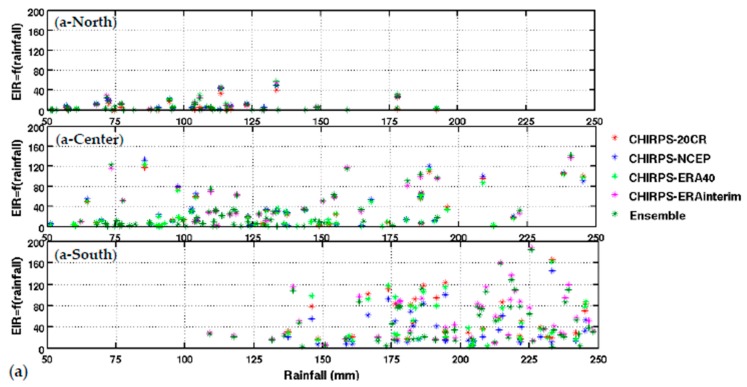

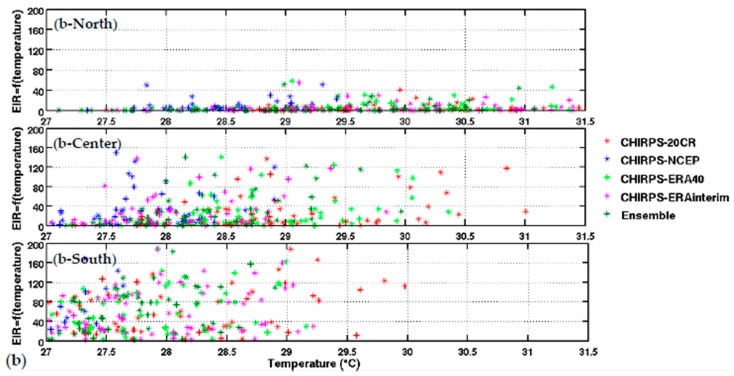
Upper panel: (**a**) variation of EIR as a function of rainfall by averaged Senegal zones (North, central, and South. Lower panel: (**b**) variation of EIR as a function of temperature by averaged Senegal zones (North, central, and South). The rainfall is from CHIRPS, and the temperature is derived from different reanalysis data. Simulated EIR are shown for the common period 1981–2001.

We now inter-compare the evolution of CHIRPS rainfall with meteorological station data and reanalysis data. The comparison is carried out for the whole country (Figure 2d–f) and for different regions (Figure 3 and Figure 4). The CHIRPS rainfall is consistent with the observed rainfall seasonal cycle derived from the averaged meteorological stations. The 20th CR shows the warmer seasonal cycle of temperature with respect to other climatic datasets (Figure 2e).

Based on regional averages, there is a clear relationship between the magnitude of malaria burden and the latitudinal gradient of the climate variables (Figure 3). The wettest stations in the South and Southeast show the largest number of malaria cases (Figure 3). A lag of two months is generally observed between the peak of the rainy season and the peak in malaria cases for all stations consistent with the results shown by [64]. A more detailed analysis of observed malaria cases is carried out in the following section. There are strong differences between the EIR simulated using the meteorological station data and the EIR simulated using the reanalysis data as inputs. This could be attributed to a nonlinear relationship between temperature and rainfall in the malaria model.

### 3.2. Seasonal Cycle of Observed Malaria

#### 3.2.1. Observed Malaria Cases in Senegal

In the Senegal River Valley, represented by the Saint-Louis weather station, it is known that within the vicinity of the “*Diama”* and “*Manantali*” hydraulic dams, the permanence of water bodies and vegetation are related to man-made irrigation schemes [65]. These man-made environmental changes have led to significant changes in the distribution of malaria vectors over this region [66,67,68,69]. Very few malaria cases are reported for Saint-Louis (Figure 2c, yellow line). The maximum in malaria cases is observed in October. The simulated EIR using CHIRPS rainfall and different reanalysis temperature datasets as inputs (Figure 2b) are qualitatively consistent with the observed malaria cases (Figure 2b–d). The low rainfall amounts explain the lowest values for most malaria parameters in the North and Northeast, compared to other stations located in the South and Southeast of the country. 

Two months of lag is observed between the peak of rainfall and the maximum number of malaria cases (Figure 2c,d). This two months of lag is related to the vector development cycle, the time for this mosquito to become infectious, the time it infects a healthy host, and the time required to incubate the parasite in humans before they show any clinical symptoms of malaria. This high malaria transmission season usually takes place in September through November in Senegal, and corresponds to the second peak of temperature.

In the northeastern part of Senegal (Ferlo), malaria transmission occurs towards the end of the rainy season as in the rest of the country (see Louga on Figure 2c). However, in some local areas with lowlands and temporal ponds, malaria transmission season can extend beyond the rainy season during very wet years [70,71]. Therefore, the EIR is highly variable and strongly depends on the location. The EIR can reach 100 infectious bites per person and per year in the upper valley of Ferlo [72,73]. Moderate values of malaria cases are shown during and just after the rainy season, with a maximum of about 100 cases in October. Although the dry season is characterized by low values of malaria cases, high transmission is always observed towards the end of the rainy season. In October, climate characteristics such as high temperature and humidity, but also important vegetation cover, are favorable toward the development of mosquito vectors. Towards the end of the rainy season, the water bodies and mature vectors are available [74]. In the typical humid savannah area of Ferlo, the seasonal transmission increases and can reach more than 100 infectious bites per person per day [75].

Dakar and Thies include more than half of the total urban population living in Senegal. The high urban population density in Dakar is caused by the important economic migration of people from rural areas [76]. Dakar, the capital of Senegal, represents the administrative and economic core of the country. Illegal occupation of the space and urbanization became uncontrolled; this disturbed the normal evacuation of water bodies in the city. This is mainly why, during the rainy season, rainfall events can cause large floods as well as the proliferation of mosquitoes. The lack of public health facilities creates inequality in access to primary care [76]. On the environmental side, liquid waste management is a key factor in urban malaria during the transmission season. Domestic wastewater is usually discharged in the vicinity of houses [76]. Some neighborhoods are invaded by those wastewaters, and the resulting man-made breeding sites favor *Anopheles* spp. mosquito development [77,78]. The black curve in Figure 2c shows the mean seasonal cycle of malaria cases in Dakar, with one of the largest peaks of malaria cases in the country. Malaria transmission risk can extend beyond the rainy season in floodplains known as *Niayes* [79]. A two month lag between the maximum of rainfall and the maximum of malaria cases is also depicted for this region.

The Kaolack region has a typical sandy and hydromorphic soil that easily promotes breeding sites during the rainy season. The maximum in malaria cases is shown in October due to a large hatching of mosquitoes following the heavy rains in August. The risk of malaria transmission is strongly related to rainfall, with very few malaria cases reported during the dry season.

In the eastern Senegal region (Tambacounda), there are several alluvial valleys within the Faleme river basin. The vegetation is abundant and varied, and implies the economic development of this region. Tambacounda is the second region where malaria transmission is the highest according to [80]. The EIR is estimated to range between 220 and 270 infectious bites per person per day during the period of high transmission. Malaria cases are observed during the early dry season due to the persistence of water bodies that can last until the late dry season. The brown curve on Figure 2c illustrates the seasonal cycle of malaria transmission in the region of Tambacounda. Malaria cases are observed from June, corresponding to the first month of rains, but the evolution is not so important at that moment because the breeding sites are not yet developed.

The *Basse Casamance* is the wettest region; its environment is characterized by important vegetation cover. The malaria transmission is very high in this area. The orange and red curves in Figure 2c show the variation of malaria for Ziguinchor and Kolda, respectively. The largest number of malaria cases is shown for both regions, and this is consistent with rainfall events affecting the region in terms of length and intensity. This maximum in malaria transmission is also due to the strengthening of favoring environmental conditions, such as vegetation cover, during this period. However, there are large discrepancies in malaria transmission in different districts of the same region. These disparities are mainly related to specific local land features, such as paddy fields, mangroves, ponds, and lowlands. The limited availability of public health services and the distance from remote rural areas to hospitals are also important parameters impacting malaria risk in that region.

#### 3.2.2. Evaluation of Malaria Simulations

The distribution of malaria for the whole country (Figure 2) and in the different Senegalese stations (Figure 3) is further analyzed in this section. The temperature and rainfall seasonal cycle difference between stations is important for the simulated malaria outcome. Higher simulated malaria parameters are shown in southern and southeastern areas, such as Ziguinchor, Kolda, and Tambacounda. For these stations, the maximum of the simulated EIR corresponds to the maximum of observed malaria cases in October. The disparities in malaria patterns between the northern and southern stations of Senegal, but also in terms of seasonality, are consistent with findings by [38]. Reference [38] evaluated the past and present impact of seasonal and interannual climate variability on malaria transmission. The findings showed that the malaria transmission belt reaches its top northward location (about 15° N) in September through November and there is a significant phase shift between rainfall and malaria seasonality. This study highlighted that large malaria transmission is generally conditioned by climatic conditions occurring two months before. These findings are also consistent with results based on field observations in Senegal, but also in Niger [81]. Other modelling approaches show that the northern epidemic fringe of malaria is more likely to spread to 17 ° N [82]. In order to characterize the capability of malaria simulations driven by reanalysis data in reproducing the observed findings, the annual cycle of observed malaria cases over Senegal is represented together with the averaged value given by each reanalysis dataset for temperature (Figure 2e) and simulated EIR (Figure 2f). The peak in malaria cases coincides with the peak of all simulated malaria parameters (October), roughly two months after the heavy rains of August, and this also occurs during the secondary temperature maximum. Figure 3a represents the annual cycle of rainfall, temperature, and simulated EIR, based on LMM outputs driven by different combinations of meteorological and CHIRPS/reanalysis data. Results are obtained by averaging the values over northern (column 1), central (column 2) and southern Senegal (column 3) for the common period 1981–2001. The simulated EIR, using reanalysis temperature and CHIRPS rainfall as drivers, is overestimated in the central and southern stations. This difference is due to the sensitivity of the LMM to the amplitudes of temperature values. These discrepancies between reanalysis datasets were also found by [83,84]. In order to further analyze variability in seasonal climate and simulated EIR, we now examine standard deviations on a per month basis in Figure 3b. In the northern domain, standard deviation exceeds the mean, indicating strong interannual variability in simulated malaria parameters. For the other stations, the standard deviation is of similar magnitude to the mean. In the southern domain, the variability is half the mean. Although the representation of the malaria seasonal cycle differs when comparing the different reanalysis-driven simulations, the trends and timing are consistent. Discrepancies are found in the magnitude of the EIR seasonal cycle, and they are related to different bias in the representation of temperature in the reanalysis datasets. Figure 4 represents the variation of EIR as a function of rainfall and temperature in Senegal from 1981 to 2001 and for different domains. The rainfall amounts required in the North are lower for the same value of EIR than in the South. Higher values of EIR are related to larger rainfall in the South. Conversely, temperatures in the South are lower than in the North, where temperatures can reach up to more than 30 °C. Regarding temperatures, NCEP temperatures are lower than the other reanalysis products. With the 20CR reanalysis, an EIR is produced up to temperatures higher than 30 °C in the centre and southern stations. Larger EIR values are simulated using ERA Interim and ERA40 reanalysis as driving conditions. Figure 2b illustrates that the simulated optimal temperature for malaria transmission lies between 27 °C and 29 °C, which is the typical temperature observed over the central and southern parts of Senegal [34,35,36]. Higher temperatures tend to increase the number of blood meals, thus further increasing the number of eggs laid by female anopheles. Very high temperatures limit mosquito vectors’ survival and parasite development. There is also a minimum temperature threshold below which the parasite cannot develop in the vector. Theoretically, if the temperature becomes very high, the conditions become unfavourable for malaria parasites, so, it is likely due to rainfall that an EIR is produced with a temperature higher than 30 °C.

These high temperature thresholds are still under debate; this is also why there are so many mosquito survival schemes available in the literature. Considering the relationship between EIR and rainfall solely, the density of scattered points is dense for low EIR and low rainfall values over northern Senegal (between 50 mm and 125 mm, see Figure 4a-North). For the central domain, the density of points is important for moderate values of rainfall (up to 175 mm, see Figure 4a-center). For the southern domain, the density of points is large for high rainfall values (up to 250 mm, see Figure 4a-South). For the central and southern domains, the density of points is larger for high EIR and moderate temperature values. This implies that malaria parameters are more related to rainfall than to temperature in the southern part of Senegal.

#### 3.3.3. Interannual and Long-Term Context

**Figure 5:**
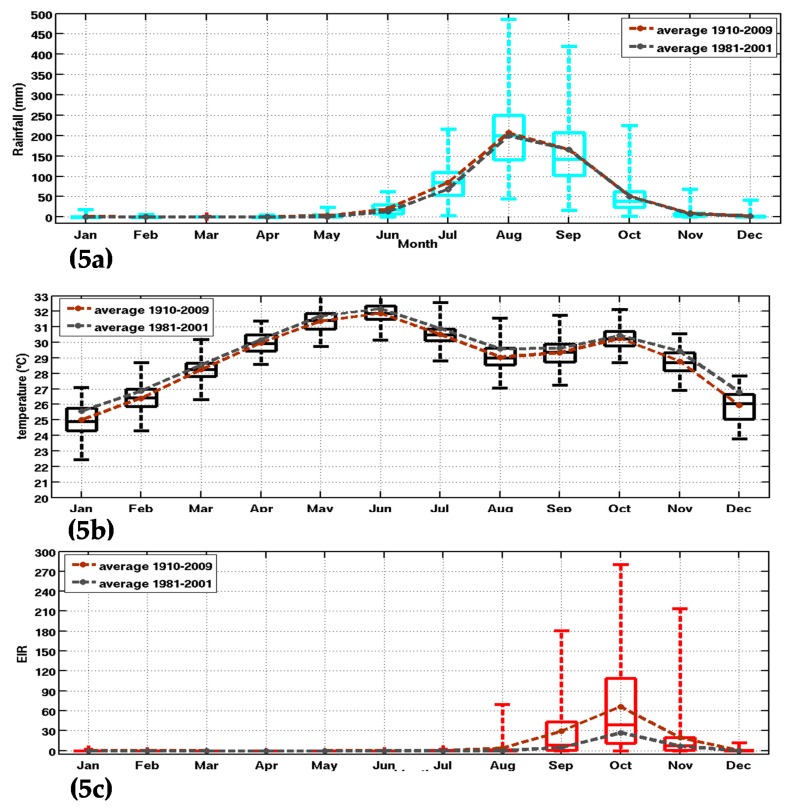
Box-and-whisker plot of climate parameters and the simulated malaria parameter: (**a**) rainfall (mm); (**b**) temperature (°C); and (**c**) simulated EIR (infectious bites per person per month) in Senegal. The whiskers and max/min outliers are shown for inputs and outputs of the Liverpool Malaria Model (LMM) for the 20th CR dataset (1910–2010). The boxes mark the median, the 25th, and the 75th percentile, while the whiskers give the minimum and maximum values. The brown and grey curves represent, respectively, the superimposed mean annual cycles on the Box-and-whisker for 1910–2009 and the common climatological period to the other reanalysis dataset (1981–2001).

An important aspect deals with the longer-term variability of the simulated malaria parameters. This is critical to better understanding the causes that produce anomalous climate-related malaria outbreaks. The interannual and seasonal variability is well-reproduced by malaria simulations driven by the 20th CR dataset (Figure 5). Consequently, we focus on this data to further characterize interannual and long-term changes in simulated EIR.

The full time period of the 20th CR was selected to analyze simulated seasonal cycle and variability. The malaria parameters show a maximum between September and November, while the rainfall peaks between July and September (Figure 5a,c). In Figure 5, the brown and grey curves, respectively, represent the mean annual cycles of the three parameters (rainfall, temperature and EIR) for 1910–2009 and 1980–2001. This is carried out to characterize multi-decadal changes in climatic parameters and simulated malaria burden for Senegal. For rainfall, slightly lower values are observed for 1980–2001 with respect to the long-term mean. The opposite is observed for temperature, with larger temperatures observed during the late period, consistently with climate change effects. The same difference in rainfall is also found for the EIR parameter, where the mean annual cycle of EIR is lower for the period 1981–2001. This is likely due to reduced rainfall during the common and short period, which includes the drought period in Sahel. The bimodal evolution of temperature peaks in May and October [62].

The simulated seasonality of malaria over Senegal is well-represented, with larger interannual variability in October as shown by a larger standard deviation (Figure 5c). Nevertheless, this interannual variability is underestimated with respect to malaria cases observations for the northern part of Senegal.

**Figure 6:**
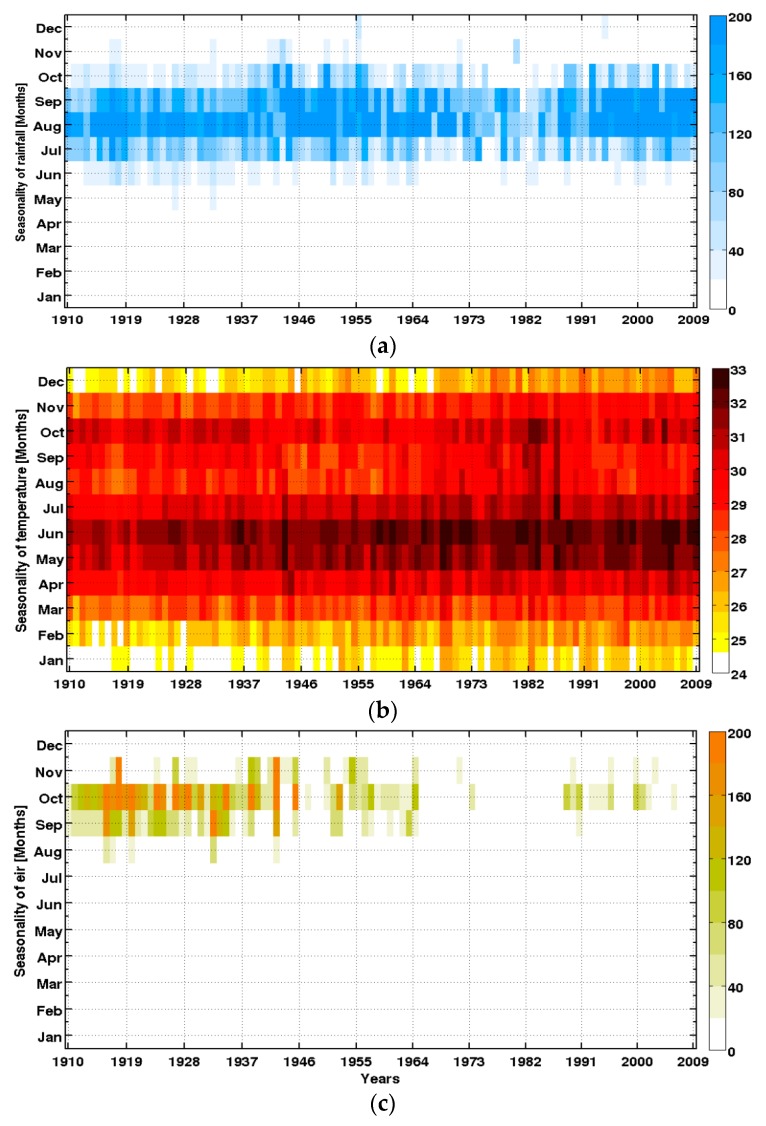
Intra- and interannual variability of climate parameters and the simulated malaria parameter: (**a**) rainfall (mm); (**b**) temperature (°C); and (**c**) simulated EIR (infectious mosquito bites per person per month), in Senegal (1910–2009).

A marked decadal variability of rainfall is observed (Figure 6). It is characterized by three phases from 1950 to the present: (i) an excess rainfall period until near the late 1960s; (ii) a period of catastrophic drought in the 1970s and 1980s; and (iii) a recovery period in the late 1990s up to today. This return to normal conditions is characterized by an increase in daily totals, while the frequency and distribution of rainfall events of recent years are increasingly variable (data not shown).

These results are consistent with findings from the Fifth Assessment Report (AR5) of the Intergovernmental Panel on Climate Change [85], which highlighted that the Sahel region suffered changes due to natural and anthropogenic forcing. Nevertheless, there is still a large debate about the impact of anthropogenic climate change on decadal to multi-decadal rainfall variability over the Sahel [86,87,88]. These changes have altered the distribution of rainfall, with significant impacts on the functioning and productivity of agro-pastoral and natural ecosystems, modifying the distribution of vector-borne diseases [28,29]. Rainfall over the Sahel presents large decadal and interannual variability. The decadal signal is modulated by anthropogenic factors, but also by large modes of natural variability, such as the Atlantic Multidecadal Oscillation. The interannual signal is mainly affected by the El Niño Southern Oscillation, the Tropical Atlantic basin, and internal atmospheric variability over the region.

Temperature is well-reproduced by the 20CR data, including its spatial distribution, its seasonal cycle, and the significant increasing trend during the recent period (Figure 6b).

The EIR seems to follow the three main rainfall phases in the Sahel and Senegal particularly (Figure 6c). The reduction in simulated EIR during the 1970–1989 drought is consistent with an observed decrease in vector abundance (in particular for *An. funestus*) as reported by field studies [17]. The LMM is more sensitive to rainfall because temperatures are generally conducive for malaria in Senegal almost all year round.

## 4. Discussion

The present study investigated the relationship between climatic parameters and simulated malaria burden over Senegal. To this aim, a malaria model fed by meteorological data was used to perform simulations that were validated with clinical observations. We used the Liverpool Malaria Model (LMM), and simulated EIR was the parameter used for validation. The model was fed by temperature based on different reanalysis data and CHIRPS rainfall, but also with rainfall from meteorological stations for validation. The simulated malaria parameters were compared to the associated rainfall and temperature seasonal cycles for each particular station and by aggregating the results along three main Senegalese regions (northern, central and southern regions). In general, there is an agreement between the different available reanalysis and meteorological observations data sets. A robust lag of two months is shown between the peak of the rainy season and the simulated peak of the malaria season for all driving climate datasets. Apart from the agreement between observed and simulated malaria parameters, some important discrepancies are also found. The LMM largely underestimates malaria burden in the North of Senegal. The LMM generally pushes the epidemic belt (characterized by low mean and large standard deviation of the EIR from year to year) too far South. The LMM simulates lower values for the northern part of Senegal, especially when using ERA40 as driving conditions, as the rain belt does not extend far enough North over the region for this dataset. Some discrepancies between different reanalyses in terms of temperature are also reflected in the malaria model outputs.

## 5. Conclusions

The risk of malaria transmission in Senegal is modulated by climate patterns, including the amount and intensity of rainfall, consistently with [89]. Wet years are very often related to high malaria burden because rainfall promotes the multiplication of breeding sites for anopheles mosquitoes. However, it is known that different anopheles mosquitoes use different types of water bodies to breed [90,91]. Rainfall also affects malaria transmission in an indirect way, because it increases the relative humidity and generally decreases temperature. For all Senegal stations, there is a long dry season, so anopheles populations are rarely developed throughout this season. The rate and amount of rainfall are key factors that determine the abundance of mosquitoes and the length of the malaria transmission season for the different sites under study [92].

The same methodology can be extended to the whole Sahelian region. Other malaria models, such as the VECtor-borne disease community model of ICTP, TRIeste (VECTRI), can also be employed to model malaria for different regions. ICTP is The Abdus Salam International Centre for Theoretical Physics, it is an international research institute for physical and mathematical sciences that operates under a tripartite agreement between the Italian Government, United Nations Educational, Scientific and Cultural Organization (UNESCO), and International Atomic Energy Agency (IAEA). Future studies should include multi-malaria model analysis to have a better assessment of malaria interannual variability over Senegal. This work can ultimately lead to the development of an early warning system for malaria risk in Senegal, once coupled to operational seasonal climate forecasts.

## Figures and Tables

**Table 1:** Datasets and stations. This table summarizes the available datasets, time periods, the spatial resolution of gridded data, and the coordinates of the different stations.

Observations														
Datasets	Period		Stations											
Malaria cases	2001–2016		Dakar 14.73° N, 17.5° W	Diourbel 14.4° N, 16.6° W	Fatick 14.21° N, 16.35° W	Kaolack 14.13° N, 16.07° W	Kolda 14.88° N, 14.08° W	Louga 15.37° N, 16.13° W		Matam 15.65° N, 13.25° W	Saint-Louis 16.05° N, 16.45° W	Tambacounda 13.77° N, 13.68° W	Thies 14.8° N, 17° W	Ziguinchor 12.55° N, 16.27° W
Meteorological and CHIRPS rainfall	1981–2010													
**Reanalysis Inputs**														
**Datasets**		**Period**							**Grid**					
20th Century Reanalysis Project daily averages [56]		1910–2009							2.5° × 2.5°					
NCEP [57]		1960–2013							2.5° × 2.5°					
ERA40 [58]		1958–2001							2.5° × 2.5°					
ERA Interim [59]		1979–2015							1.5° × 1.5°					

CHIRPS: Climate Hazards Group InfraRed Precipitation with Stations; NCEP: National Centers for Environmental Prediction; ERA: European Center for Medium Range Weather Forecast reanalysis.

## Supplementary Materials

The following are available online at www.mdpi.com/1660-4601/14/10/1119/s1, Table S1: LMM parameter settings.

## References

[1] Bruce-Chwatt L.J. (1985). Essential Malariology.

[2] Gupta S., Hill A.S. (1998). Dynamic interaction in malaria: Host heterogeneity meets parasite polymorphism. Proc. Soc. Lond. B.

[3] Singh B., Daneshvar C. (2010). *Plasmodium* knowlesi Malaria in Malaysia. Med. J. Malays..

[4] Sinka M.E., Bangs M.J., Manguin S., Coetzee M., Mbogo C.M., Hemingway J., Patil A.P., Temperley W.H., Gething P.W., Kabaria C.W. (2010). The dominant Anopheles vectors of human malaria in Africa, Europe and the Middle East: Occurrence data, distribution maps and bionomic précis. Parasites Vectors.

[5] Coetzee M., Craig M., Le Sueur D. (2000). Distribution of African malaria mosquitoes belonging to the *Anopheles gambiae* complex. Parasitol. Today.

[6] Fontenille D., Lochouarn L. (1999). The complexity of the malaria vectorial system in Africa. Parassitologia.

[7] Hay S.I., Okiro E.A., Gething P.W., Patil A.P., Tatem A.J., Guerra C.A., Snow R.W. (2010). Estimating the Global Clinical Burden of *Plasmodium falciparum* Malaria in 2007. PLoS Med..

[8] Worrall E., Rietveld A., Delacollette C. (2014). The burden of malaria epidemics and cost-effectiveness of interventions in epidemic situation in Africa. Am. J. Trop. Med. Hyg..

[9] McMichael A.J., Sloof R.H., Kovats S. (1996). Changement Climatique et Santé Humaine—Risques et Mesures à Prendre, Contribution in WHO.

[10] Thomson M.C., Doblas-Reyes F.J., Mason S.J., Hagedorn R., Connor S.J., Phindela T., Morse A.P., Palmer T.N. (2006). Malaria early warning based on seasonal climate forecasts from multi-model ensembles. Nature.

[11] Sachs J., Malaney P. (2002). The economic and social burden of malaria. Nature.

[12] World Health Organization (2005). The World Health Report 2005: Make Every Mother and Child Count.

[13] Hulme M. (1992). Rainfall changes in Africa: 1931–1960 to 1961–1990. Int. J. Climatol..

[14] Folland C.K., Palmer T.N., Parker D.E. (1986). Sahel rainfall and worlwide sea temperature 1901–1985. Nature.

[15] Dai A., Lamb P.J., Trenberth K.E., Hulme M., Jones P.D., Xie P. (2004). The recent Sahel drought is real. Int. J. Climatol..

[16] Lebel T., Ali A. (2009). Recent trends in the Central and Western Sahel rainfall regime 1990–2007. J. Hydrol..

[17] Mouchet J., Faye O., Julvez J., Manguin S. (1996). Drought and malaria retreat in the Sahel, West Africa. Lancet.

[18] Faye O., Fontenille D., Herve J.P., Diack P.A., Diallo S., Mouchet J. (1993). Le paludisme en zone sahélienne du Sénégal: Données entomologiques sur la transmission. Ann. Soc. Belg. Med. Trop..

[19] Rogier C., Trape J.F. (1993). Malaria attacks in children exposed to high transmission: Who is protected?. Trans. R. Soc. Trop. Med. Hyg..

[20] Carnevale P., Robert V., Molez J.F., Baudon D. (1984). Faciès épidémiologique des paludismes en Afrique sub-saharienne. Etudes Med..

[21] Doumbo O., Sangare O., Touré Y. (1989). Malaria in the Sahel: The Example of Mali. Tropical Transmissible Diseases.

[22] Faye O., Gaye O., Fontenille D., Hébrard G., Konate L., Sy N., Herve J.P., Toure Y.T., Diallo S., Molez J.F. (1995). Malaria decrease and drought in the *Niayes* area of northwestern Senegal. Cah. Sante.

[23] Pagès F., Texier G., Pradines B. (2008). Malaria transmission in Dakar: A two-year survey. Malar. J..

[24] Lehmann T., Dao A., Yaro A.S., Adamou A., Kassogue Y., Diallo M., Sékou T., Coscaron-Arias C. (2010). Aestivation of the African Malaria Mosquito, *Anopheles gambiae* in the Sahel. Am. J. Trop. Med. Hyg..

[25] Fontenille D., Lochouarn L., Diagne N., Sokhna C., Lemasson J.J., Diatta M., Konate L., Faye F., Rogier C., Trape J.F. (1997). High annual and seasonal variations in malaria transmission by anophelines and vector species composition in Dielmo, a holoendemic area in Senegal. Am. J. Trop. Med. Hyg..

[26] Scott T.W., Takken W. (2012). Feeding strategies of anthropophilic mosquitoes result in increased risk of pathogen transmission. Trends Parasitol..

[27] Koella J.C., Sørensen F.L., Anderson R.A. (1998). The malaria parasite, *Plasmodium* falciparum, increases the frequency of multiple feeding of its mosquito vector, Anopheles gambiae. Proc. Boil. Sci..

[28] Nájera J.A., Kouznetsov R.L., Delacollette C. (1998). Malaria Epidemics: Detection and Control, Forecasting and Prevention.

[29] Badara S., Dia I., Konate L., Ayala D., Fontenille D., Cohuet A. (2012). Population genetic structure of the malaria vector Anopheles funestus, in a recently re-colonized area of the Senegal River basin and human-induced environmental changes. Parasites Vectors.

[30] Grover-Kopec E.K., Blumenthal M.B., Ceccato P., Dinku T., Omumbo J.A., Connor S.J. (2006). Web-based climate information resources for malaria control in Africa. Malaria J..

[31] Ndiaye F., Molez J.F., Trape J.F., Delaunay V. (1998). Endémie palustre. La Situation Démographique et Épidémiologique Dans la Zone de Niakhar au Senegal: 1984–1996.

[32] Fontenille D., Lochouarn L., Diatta M., Sokhna C., Dia I., Diagne N., Lemasson J.J., Ba K., Tall A., Rogier C. (1997). Four years’ entomological study of the transmission of seasonal malaria in Senegal and the bionomics of *Anopheles gambiae* and A. arabiensis. Trans. R. Soc. Trop. Med. Hyg..

[33] Freeman T., Bradley M. (1996). Temperature is predictive of severe malaria years in Zimbabwe. Trans. R. Soc. Trop. Med. Hyg..

[34] Patz J.A., Olson S.H. (2006). Malaria risk and temperature: Influences from global climate change and local land use practices. Proc. Natl. Acad. Sci. USA.

[35] Murdock C.C., Paaijmans K.P., Bell A.S., King J.G., Hillyer J.F., Read A.F., Thomas M.B. (2012). Complex effects of temperature on mosquito immune function. Proc. R. Soc. B.

[36] Alonso D., Menno M.J., Pascual M. (2011). Epidemic malaria and warmer temperatures in recent decades in an East African highland. Proc. R. Soc. B.

[37] Bouma M.J., Dye C., van der Kaay H.J. (1995). El Niño Southern Oscillation as a Possible Early Warning System for Falciparum Malaria Epidemics in Northern Pakistan: Épidémiologie et de Contrôle du Paludisme Dans le Nord du Pakistan.

[38] Lindsay S.W., Birley M.H. (1996). Climate change and malaria transmission. Ann. Trop. Med. Parasitol..

[39] Tanser F.C., Sharp B., Le Sueur D. (2003). Potential effect of climate change on malaria transmission in Africa. Lancet.

[40] Besancenot J.P., Ndione J.A., Handschumacher P., Ibrahima M., Laaidi K. (2004). Climat, eau et santé au Sahel ouest-Africain. Science Changements Planétaires/Sécheresse.

[41] Ndione J.A., Diop M., Lacaux J.P., Gaye A.T. (2008). Variabilitée intrasaisonnière de la Pluviomètrie et émergence de la fièvre de la vallée du rift (FVR) dans la vallée du fleuve Sénégal: Nouvelles considérations. Climatologie.

[42] Ndione J.A., Besancenot J.P., Lacaux J.P., Sabatier P. (2003). Environnement et épidémiologie de la fièvre de la vallée du Rift (FVR) dans le bassin inférieur du fleuve Sénéegal. Environ. Risques Sante.

[43] Caminade C., Ndione J.A., Kebe C.M.F., Jones A.E., Danuor S., Tay S., Tourre Y.M., Lacaux J.P., Vignolles C., JDuchemin J.B. (2011). Mapping Rift Valley fever and malaria risk over West Africa using climatic indicators. Atmos. Sci. Lett..

[44] Hoshen M.B., Morse A.P. (2004). A weather-driven model of malaria transmission. Malar. J..

[45] Ermert V., Fink A.H., Jones A.E., Morse A.P. (2011). Development of a new version of the Liverpool Malaria Model. I. Refining the parameter settings and mathematical formulation of basic processes based on a literature review. Malar. J..

[46] Morse A.P., Doblas-Reyes F.J., Hoshen M.B., Hagedorn R., Palmer T.N. (2005). A forecast quality assessment of an end-to-end probabilistic multi-model seasonal forecast system using a malaria model. Tellus.

[47] Jones A., Morse A. (2010). Application and validation of a seasonal ensemble prediction system using a dynamic malaria model. J. Clim..

[48] Ermert V., Fink A.H., Jones A.E., Morse A.P. (2011). Development of a new version of the Liverpool Malaria Model. II. Calibration and validation for West Africa. Malar. J..

[49] Diouf I., Deme A., Ndione J.A., Gaye A.T., Rodriguez F.B., Cisse M. (2013). Climate and health: Observation and modeling of malaria in the Ferlo (Senegal). C. R. Biol. Acad. Sci..

[50] Ermert V., Fink A.H., Morse A.P., Paeth H. (2012). The Impact of Regional Climate Change on Malaria Risk due to Greenhouse Forcing and Land-Use Changes in Tropical Africa. Res. Environ. Health Perspect..

[51] Ndione J.A. (2002). Bilan climatique de l’Observatoire ROSELT du Ferlo (Sénégal).

[52] Deuxième Communication Nationale du Sénégal sur Changements Climatiques: Vulnérabilité du Secteur de la Santé. http://unfccc.int/resource/docs/natc/sennc2.pdf.

[53] Programme Nationale de Lutte contre le Paludisme (2009). Situation de la Lutte Contre le Paludisme au Sénégal. Enquête Nationale sur le Paludisme au Sénégal Entre 2008 et 2009 (ENPS-II).

[54] Ndiaye S., Ayad M. (2009). Enquête Nationale sur le Paludisme au Sénégal 2008–2009.

[55] Giardina F., Gosoniu L., Konate L., Diouf M.B., Perry R., Gaye O., Faye O., Vounatsou P. (2012). Estimating the burden of malaria in Senegal: Bayesian zero-inflated binomial geostatistical modeling of the MIS 2008 data. PLoS ONE.

[56] Compo G.P., Whitaker J.S., Sardeshmukh P.D., Matsui N., Allan R.J., Yin X., Gleason B.E., Vose R.S., Rutledge G., Bessemoulin P. (2011). The twentieth century reanalysis project. Q. J. R. Meteorol. Soc..

[57] Kalnay E., Kanamitsu M., Kistler R., Collins W., Deaven D., Gandin L., Joseph D. (1996). The NCEP/NCAR 40-year reanalysis project. Bull. Am. Meteorol. Soc..

[58] Uppala S.M., Kållberg P.W., Simmons A.J., Andrae U., Bechtold V., Fiorino M., Woollen J. (2005). The ERA-40 re-analysis. Q. J. R. Meteorol. Soc..

[59] Simmons A., Uppala S., Dee D., Kobayashi S. (2007). ERA-Interim: New ECMWF reanalysis products from 1989 onwards. ECMWF Newslett..

[60] US Geological Survey A Quasi-Global Precipitation Time Series for Drought Monitoring. https://pubs.usgs.gov/ds/832/pdf/ds832.pdf.

[61] Jones A.E., Morse A.P. (2012). Skill of ENSEMBLES seasonal re-forecasts for epidemic malaria prediction in West Africa. Geophys. Res. Lett..

[62] Van der Linden P.J., Mitchell J.F.B. (2009). ENSEMBLES. Climate Change and Its Impacts: Summary of Research and Results from the ENSEMBLES Project.

[63] Nicholson S.E., Grist J.P. (2001). A conceptual model for understanding rainfall variability in the West African Sahel on interannual and interdecadal timescales. Int. J. Climatol..

[64] Diouf I. (2016). Climate and Health: Observations and Modeling of Seasonal Malaria Incidence for Its Predictability over Senegal and the Sahel. Ph.D. Thesis.

[65] Ndiath M., Sarr J.B., Lobna G., Catherine M., Sougoufara S., Konate L., Remoue F., Hermann E., Trape J.F., Riveau G. (2012). Low and seasonal malaria transmission in the middle Senegal River basin: Identification and characteristics of Anopheles vectors. Parasites Vectors.

[66] Sow S., de Vlas S.J., Engels D., Gryseels B. (2002). Water-related disease patterns before and after the construction of the Diama dam in northern Senegal. Ann. Trop. Med. Parasitol..

[67] Dia I., Konate L., Samb B., Sarr J.B., Diop A., Rogerie F., Faye M., Riveau G., Remoue F., Diallo M. (2008). Bionomics of malaria vectors and relationship with malaria transmission and epidemiology in three physiographic zones in the Senegal River Basin. Acta Trop..

[68] Faye O., Diagne M., Ndiongue S., Sy M., Diack P.A. (1992). Le Paludisme Dans la Zone du Périmètre Irrigué de Diomandou; Podor. Sénégal: Résultats des enquêtes effectuées de juin 1990 à novembre 1991. Doc. Orstom Dakar.

[69] Monjour L., Richard-Lenoble D., Sidatt M., Druilhe P., Mogahed A., Gentilini M. (1982). Geographic distribution of malaria in the valley of the Senegal River: School survey (sero-immunologic evaluation, year 1973). Bull. Soc. Pathol. Exot. Ses Fil..

[70] Faye O., Gaye O., Konaté L., Molez J.F., Feller-Dansokho E., Hervé J.P. (1998). Prediction and prevention of malaria epidemics in the valley of the Senegal River. Cah. Detudes Rech. Francoph. Sante.

[71] Tourre Y.M., Lacaux J.P., Vignolles C., Ndione J.A., Lafaye M. (2008). Mapping of zones potentially occupied by mosquitoes (ZPOMs) *Aedes vexans* and *Culex poicipiles*, the main vectors of Rift Valley fever in Senegal. Geospat. Health.

[72] Lemasson J.J., Fontenille D., Lochouarn L., Dia I., Simard F. (1997). Comparison of behaviour and vector efficiency of anopheles gambiae and anopheles arabiensis (diptera: Culicidae) in Barkedji, a Sahelian area of Senegal. J. Med. Entomol..

[73] Ngom H.M., Ndione J.A., Ba Y., Konaté L., Faye O., Diallo M., Dia I. (2013). Spatio-temporal analysis of host preferences and feeding patterns of malaria vectors in the sylvo-pastoral area of Senegal: Impact of landscape classes. Parasites Vectors.

[74] Traoré L.M., Fontenille D., Diallo M., Ba Y., Zeller H.G., Mondo M., Adam F., Thonon J., Maiga A. (2001). Arbovirus surveillance from 1990 to 1995 in the Barkedji area (Ferlo) of Senegal, a possible natural focus of Rift Valley Fever virus. J. Med. Entomol..

[75] Bomblies A., Eltahir E.A. (2009). Assessment of the impact of climate shifts on malaria transmission in the Sahel. Ecohealth.

[76] Machault V., Vignolles V.C., Pagès F. (2010). Spatial heterogeneity and temporal evolution of malaria transmission risk in Dakar, Senegal, according to remotely sensed environmental data. Malar. J..

[77] MacHaultc V., Gadiaga L., Vignolles C. (2009). Highly focused anopheline breeding sites and malaria transmission in Dakar. Malar. J..

[78] Trape J.F., Lefebvre-Zante E., Legros F., Ndiaye G., Bouganali H., Druille P., Salem G. (1992). Vector Density Gradients and the Epidemiology of Urban Malaria in Dakar, Senegal. Am. J. Trop. Med. Hyg..

[79] Diallo S., Ndir O., Faye O., Diop B.M., Dieng Y. (1998). Malaria in the Southern Sanitary District of Dakar (Senegal). Parasitemia and malaria attacks. Bull. Soc. Pathol. Exot..

[80] Samb LM. (1993). Epidémiologie du Paludisme et de la Bilharziose à Wassadou, Département de Tambacounda. Ph.D. Thesis.

[81] Gianotti R.L., Bomblies A., Eltahir E.A.B. (2009). Hydrologic modeling to screen potential environmental management methods for malaria vector control in Niger. Water Resour. Res..

[82] Le Sueur D., Binka F., Lengeler C., de Savigny D., Snow R.W., Teuscher T., Touré Y.T. (1997). An atlas of malaria in Africa. Afr. Health.

[83] Manzanas R., Amekudzi L.K., Preko K., Herrera S., Gutiérrez J.M. (2014). Precipitation variability and trends in Ghana: An intercomparison of observational and reanalysis products. Clim. Chang..

[84] Michele M., Rienecker M.J., Suarez R.G., Ricardo T., Julio B., Emily L., Michael G.B., Siegfried D.S., Lawrence T., Gi-Kong K. (2011). MERRA: NASA’s modern-era retrospective analysis for research and applications. J. Clim..

[85] Smith K.R., Woodward A., Campbell-Lendrum D., Chadee D., Honda Y., Liu Q., Olwoch J., Revich B., Sauerborn R. (2014). Human health: Impacts, adaptation, and co-benefits. Part A: Global and Sectoral Aspects Contribution of Working Group II to the Fifth Assessment Report of the Intergovernmental Panel on Climate Change.

[86] Nicholson S.E., Some B., Kone B. (2000). An analysis on recent rainfall conditions in West Africa, including the rainy season of 1997 ENSO year. J. Clim..

[87] Giannini A., Saravanan R., Chang P. (2003). Oceanic forcing of Sahel rainfall on interannual to interdecadal time scales. Science.

[88] Mohino E., Janicot S., Bader J. (2011). Sahel rainfall and decadal to multi-decadal SST variability. Clim. Dyn..

[89] Koenraadt C.J.M., Githeko A.K., Takken W. (2004). The effects of rainfall and evapotranspiration on the temporal dynamics of *Anopheles gambiae* s.s. and Anopheles arabiensis in a Kenyan village. Acta Trop..

[90] Nagpal B.N., Sharma V.P. (1995). Indian Anophelines. New Delhi: Baba Barkha Nath Printers and Lebanon.

[91] Fillinger U., Sonye G., Killeen G.F., Knols B.G., Becker N. (2004). The practical importance of permanent and semipermanent habitats for controlling aquatic stages of *Anopheles gambiae* sensu lato mosquitoes: Operational observations from a rural town in Western Kenya. Trop. Med. Int. Health.

[92] Sabatier P., Fontaine B., Roucou P., Bicout D. (2008). Analysis of Climate Change and Impacts on Water and Health.

