# Supplementary material for: Comparison of Malaria Simulations Driven by Meteorological Observations and Reanalysis Products in Senegal
Source: Int J Environ Res Public Health. 2017 Sep 25;14(10):1119. doi: 10.3390/ijerph14101119 (PMC5664620; doi:10.3390/ijerph14101119)
Supplement: Supplementary file 1 [file ijerph-14-01119-s001.pdf]

International Journal of
Environmental Research and Public Health

MDPI

Supplementary Material

Comparison of Malaria Simulations Driven by Meteorological Observations and Reanalysis Products in Senegal

Ibrahima Diouf, Belen Rodriguez-Fonseca, Abdoulaye Deme, Cyril Caminade, Andrew P. Morse, Moustapha Cisse, Ibrahima Sy, Ibrahima Dia, Volker Ermert, Jacques-André Ndione and Amadou Thierno Gaye

Table S1. Review of the LMM set of parameter settings.

Sym	Parameter	Unit	Val_{2010}	R_{lit}	
D_{gH}	humid degree days of the gonotrophic cycle	degree days	37.1	37.1	37.1
D_{gL}	dry degree days of the gonotrophic cycle	degree days	65.4	65.4	65.4
T_{gH}	humid gonotrophic temperature threshold	°C	7.7	7.7	7.7
T_{gL}	dry gonotrophic temperature threshold	°C	4.5	4.5	4.5
*R_*	10-day accumulated precipitation threshold	mm	10	10	NA
R•	rainfall laying multiplier	-	1.0	NU	NA
$\#E_p$	number of produced eggs per female mosquito	eggs	NU	CA	5-290
$\#E_o$	number of oviposited eggs per female mosquito	eggs	NU	**Eq. 2**	NA
U_1	lower threshold of unsuitable rainfall conditions (fuzzy distribution model)	mm	NU	**0**	0
S	most suitable rainfall condition (fuzzy distribution model)	mm	NU	CA	NA
U_2	upper threshold of unsuitable rainfall conditions (fuzzy distribution model)	mm	NU	CA	NA
CAP	cap on the number of fertile mosquitoes	-	10,000	CA	NA
MMA	mosquito mature age	days	15	**12**	11.2–30
$\eta_{d,\neg R}$	rainfall independent immature daily mosquito survival probability	%	NU	82.5	52.7–99.9
η_d	daily immature mosquito survival probability	%	Eq. 3	**Eq. 4**	52.7–89.9
p_d	daily mosquito survival probability	%	Martens I	**Martens II**	Add. file 4
$p_{d\downarrow}$	dry season mosquito survival probability shift	%	NU	CA	Add. file 4
D_s	degree-days of the sporogonic cycle	degree days	111.0	111.0	111.0–204.4
T_s	sporogonic temperature threshold	°C	18	**16**	14.2–19.0
a	human blood index	%	50	**80**	0–100
b	mosquito-to-human transmission efficiency	%	50	**30**	1–50
$c_{a\rightarrow c}$	adult-child conversion rate	-	NU	**0.5**	0.28–0.5
HIA	human infectious age	days	14	**20**	12–30
r	daily human recovery rate	day^{-1}	0.0284	**0.0050**	0.0015–0.0385
GF	fraction of gametocyte carriers	%	NU	**50**	10–70
c	human-to-mosquito transmission efficiency	%	50	**20**	0–37.9
tr_{im}	trickle of the number of added infectious mosquitoes	-	1.01	1.01	NA

LMM model parameters and mathematical formulations with regard the new settings in the LMM2010. Columns: Sym: symbol of the model parameter; Parameter: name of the parameter; Val2010: parameter value or mathematical formulation of the LMM2010; ref2010: LMM2010 reference;

Rlit: literature values. Abbreviations: NU: not used; NA: not available; CA: will be calibrated in the second part of the study [45]. Martens II refers to Martens et al (1997).

References

Martens, W.J.M. Health impacts of climate change and ozone depletion: An eco-epidemiological modelling approach. Ph.D. Thesis, Maastricht University, Maastricht, The Netherlands, 1997.
